# Glucose >200 mg/dL during Continuous Glucose Monitoring Identifies Adult Patients at Risk for Development of Cystic Fibrosis Related Diabetes

**DOI:** 10.1155/2016/1527932

**Published:** 2016-11-24

**Authors:** J. L. Taylor-Cousar, J. S. Janssen, A. Wilson, C. G. St. Clair, K. M. Pickard, M. C. Jones, S. J. Brayshaw, C. S. Chacon, C. M. Barboa, M. K. Sontag, F. J. Accurso, D. P. Nichols, M. T. Saavedra, J. A. Nick

**Affiliations:** ^1^Department of Medicine, National Jewish Health, 1400 Jackson Street, Denver, CO 80206, USA; ^2^Pediatrics, National Jewish Health, 1400 Jackson Street, Denver, CO 80206, USA; ^3^Department of Pediatrics, University of Colorado School of Medicine and Children's Hospital Colorado, 13123 E 16th Ave, Aurora, CO 80045, USA; ^4^Colorado School of Public Health and University of Colorado, 13001 East 17th Place, Campus Box B119, Aurora, CO 80045, USA

## Abstract

*Rationale*. Cystic fibrosis related diabetes (CFRD) is the most common comorbidity in patients with CF. In spite of increased screening, diagnosis, and treatment of CFRD, the mortality rate in patients with CFRD still far exceeds the mortality rate in those without CFRD. Guidelines suggest that screening for CFRD be performed annually using the 2-hour 75-gram oral glucose tolerance test (OGTT). Adherence to recommended screening has been poor, with only approximately one-quarter of adults with CF undergoing OGTT in 2014. Use of continuous glucose monitoring (CGM) for diagnosis may become an alternative.* Objectives*. Our objective was to determine whether abnormal CGM predicts subsequent development of CFRD, lung function, and body mass index (BMI) decline and increased rate of CF pulmonary exacerbations in adults with CF.* Methods*. In a prospective single center pilot trial from September 2009 to September 2010, 21 adult patients due for routine OGTT were recruited to complete simultaneous 3-day CGM and 2-hour 75 gram OGTT. Subsequently, clinical information was reviewed from 2008 to 2015.* Conclusions*. There was a moderate correlation between interpreted results of 2-hour OGTT and CGM (*p* = 0.03); CGM indicated a greater level of glucose impairment than OGTT. Glucose >200 mg/dL by CGM predicted development of CFRD (*p* = 0.0002).

## 1. Introduction

Advances in treatment have led to an increase in survival in people with cystic fibrosis (CF). As patients age with CF, they experience an increased rate of complications, including cystic fibrosis related diabetes (CFRD) [1]. CFRD results from the progressive inflammatory destruction of the pancreas [2, 3] and leads to the development of insulin insufficiency with varying levels of insulin resistance during acute illness [4]. The ferret CF model also shows that early inflammation leads to pancreatic cell destruction and replacement with fibrosis [5]. These pathologic changes correspond to significant dysregulation of blood glucose and insulin. Thus, CFRD shares features with both type I and type II diabetes [4]. While patients with CF have not been observed to develop macrovascular complications of diabetes, microvascular complications are seen [6]. Importantly, diagnosis of CFRD is associated with poor nutrition status and decreased lung function and survival [7–11].

Recommendations for diagnosis, screening, and management of CFRD were written in a jointly created clinical care guideline by The Cystic Fibrosis Foundation (CFF), the American Diabetes Association (ADA), and the Pediatric Endocrine Society (PES) [12]. Based on the guideline, screening for CFRD should be performed annually on patients with CF >10 years of age using the 2-hour 75-gram oral glucose tolerance test (OGTT). Patients with 2 h OGTT plasma glucose >200 mg/dL meet criteria for the diagnosis of CFRD.

Because of the time-consuming nature of the test, and requirement that patients fast prior to testing, adherence to the guidelines has been challenging; approximately one-quarter of adults with CF were tested in 2014 [1]. This result suggests that an alternative means of evaluation is needed for the timely diagnosis of CFRD. One such option may be continuous glucose monitoring (CGM). Although infrequently used in US CF Centers, CGM has been validated in children and adolescents with CF [13, 14]. Additionally, two small studies showed that early glucose abnormalities are detectable by CGM in children and adults with CF and are associated with historical decline and contemporaneously worse health outcomes [15, 16]. One study in children with CF <18 years of age showed that abnormal CGM was the strongest predictor of abnormal glucose metabolism at 2.5-year follow-up [17]. However, these studies did not answer the question of whether abnormal CGM predicts future health outcomes in adults with CF.

We hypothesized that abnormal CGM results would be associated with deterioration in health. Our objectives for this study were to determine whether abnormal CGM results are associated with subsequent development of CFRD, lung function, and BMI decline and increased rate of CF pulmonary exacerbations.

## 2. Materials and Methods

### 2.1. Study Design

To evaluate the correlation between CGM and OGTT for detection of CFRD in adult patients with CF, we conducted a prospective single center trial in 2009-2010 in which 21 adult patients due for routine OGTT underwent simultaneous 3-day CGM and 2-hour 75 g OGTT [18]. Subsequently, to determine whether abnormal CGM predicted health decline, we performed a retrospective observational study. Medical records and data from medical charts and the CFF registry were reviewed from 2008 to 2015 to obtain clinical information (diagnosis of CFRD, pulmonary function, BMI, and pulmonary exacerbations). The original protocol was approved by the National Jewish Health Institutional Review Board (NJH IRB), and subjects provided written informed consent. Prior to the conduct of the retrospective review, the new proposal underwent expedited review and approval by the NJH IRB.

### 2.2. End Points

OGTTs were conducted using the 2-hour 75-gram oral glucose tolerance test. For OGTT, the following definitions were used: fasting glucose >126 mg/dL or 2-hour glucose >200 mg/dL indicated CFRD, fasting glucose 100–125 mg/dL indicated impaired fasting glucose (IFG), and 2-hour glucose 140–199 mg/dL indicated impaired glucose tolerance (IGT) [12, 19]. In order to* explore *the correlation between 1-hour OGTT glucose levels and CGM results, 1-hour OGTT results were also interpreted using the 2-hour OGTT definitions. For subjects without diabetes who did not have OGTT in the observation period (*n* = 5/10), the alternative* guideline* criteria were used to determine the development of CFRD: hemoglobin A1C (A1C) ≥6.5% or RPG ≥200 mg/dL in conjunction with polyuria and polydipsia [12]. One Touch® glucometer and Medtronic CGM® system were used to obtain CGM data. A board-certified endocrinologist (JJ) who was blinded to the OGTT results interpreted CGM data. Although there are no officially defined criteria for interpreting CGM, for our pilot study,* a priori* we used the American Diabetes Association criteria for OGTT [19] to define the following criteria to interpret CGM, all values were required on two separate dates: fasting glucose >126 mg/dL or random glucose >200 mg/dL indicated CFRD; random glucose >140 mg/dL indicated IGT; fasting glucose >100 mg/dL indicated IFG.

Each subject's highest lung function and BMI for the year prior to OGTT/CGM testing, and for each of the 5 years following OGTT/CGM testing, were extracted from the CF registry. The number of exacerbations each subject had for each of the 5 years following OGTT/CGM testing was also extracted from the CF registry, and the average number of exacerbations per year was calculated. During the period of retrospective review, three subjects died and three patients moved away from the center; all available data points were included for these subjects. Six patients began chronic use of Cystic Fibrosis Transmembrane Conductance Regulator (CFTR) modulators during the period of retrospective review (two patients began ivacaftor, and four patients began lumacaftor/ivacaftor). The highest FEV_1_ and BMI for the year prior to initiation of CFTR modulators were used for analysis of decline in these parameters over time.

### 2.3. Statistical Analysis

Means and standard deviations are reported for demographic information (Graph Pad Prism v6.07). Agreement between test methods (OGTT and CGM) was evaluated using both Pearson correlation and the Bland-Altman method. (Graph Pad Prism v6.07). Log-rank (Mantel-Cox) test was used to evaluate whether abnormal CGM predicts subsequent development of CFRD. Using two-sample *t*-tests, average lung function, BMI, and average exacerbation rate were compared between subjects who developed CFRD and those who did not develop CFRD. Standard error of the mean is reported for group means compared by *t*-tests. Lung function and BMI decline over time were assessed using simple linear regression. Pearson correlation was calculated to evaluate the relationship between maximum CGM and lung function and BMI decline. *p* values < 0.05 were considered significant.

## 3. Results

### 3.1. Subjects

Twenty-one subjects were enrolled. Seventy-six percent (16/21) of subjects were female. The average age of the subjects was 32.4 years (SD 13.1; median 27.0, range 20–65). Fifty-two percent (11/21) of subjects were homozygous for the F508del mutation; 90% (19/21) of subjects had at least one copy of the F508del mutation. All patients were pancreatic insufficient. Average percent of predicted FEV_1_ and average BMI of subjects at the time of enrollment was 68.8 (SD 5.5; range, 22–109) and 21.6 (range 17.1–29.1; SD 0.72), respectively (Table 1).

### 3.2. Categorization of Glucose Tolerance

**Figure 1:**
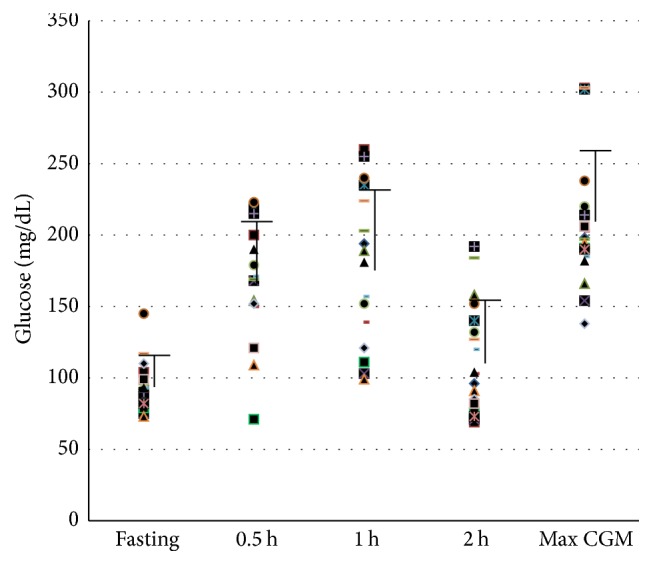
Average and individual glucose values by test method. Individual subject glucose values for initial study OGTT at each time point measured and maximum glucose value obtained by CGM are shown. Error bars represent average subject values for each OGTT time point and average maximum CGM value.

**Figure 2:**
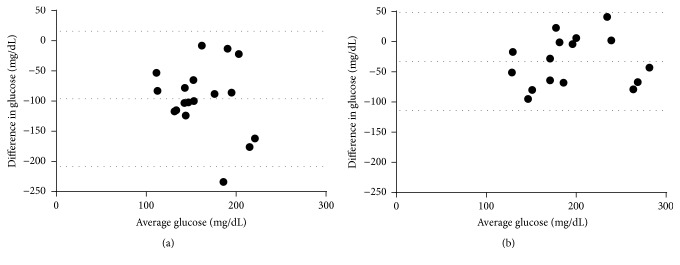
Bland-Altman evaluation of agreement between glucose testing methods. (a) Two-hour OGTT values compared to peak CGM values. (b) One-hour OGTT values compared to peak CGM values. Dotted lines indicate bias and 95% limits of agreement.

One patient did not have adequate data for OGTT interpretation due to improper collection of the timed samples. Two patients did not have adequate data for interpretation by CGM due to failure of the device and/or data retrieval. AIC was ≤5.9% for all 18 patients with complete data. For these 18 patients (mean results for test methods shown in Figure 1), there was a modest correlation between interpreted results from OGTT and CGM (*r* = 0.52, 95% CI 0.07 to 0.79, *p* = 0.03; not shown); only 3/18 (17%) comparisons yielded the same result (two subjects with impaired glucose tolerance and one subject with CFRD). In 14 of 15 (93%) subjects for whom results differed between the two tests, CGM indicated a greater level of impairment than OGTT.* One-hour* OGTT glucose interpretation yielded the same result as that of CGM in 11/17 (65%) subjects. There was a strong positive correlation between one-hour OGTT interpretation and that of CGM (*r* = 0.77, 95% CI 0.46 to 0.91, *p* = 0.0003; not shown). In 5/6 (83%) of the subjects for whom results differed between the two tests, CGM indicated a greater level of impairment. We also compared maximum CGM glucose to blood glucose based on 1-hour and 2-hour OGTT using the Bland-Altman method (Figure 2). The comparison between OGTT and maximum CGM values showed poor agreement between the two methods (bias −96.06, SD 56.91, 95% limit of agreement −207.6 to 15.49). In contrast, the comparison between* 1-hour *OGTT and maximum CGM values showed some agreement (bias −32.81, SD 41.18, 95% limit of agreement −113.5 to 47.9).

### 3.3. Impact of Abnormal CGM on Subsequent Health

#### 3.3.1. Diagnosis of CFRD

**Figure 3:**
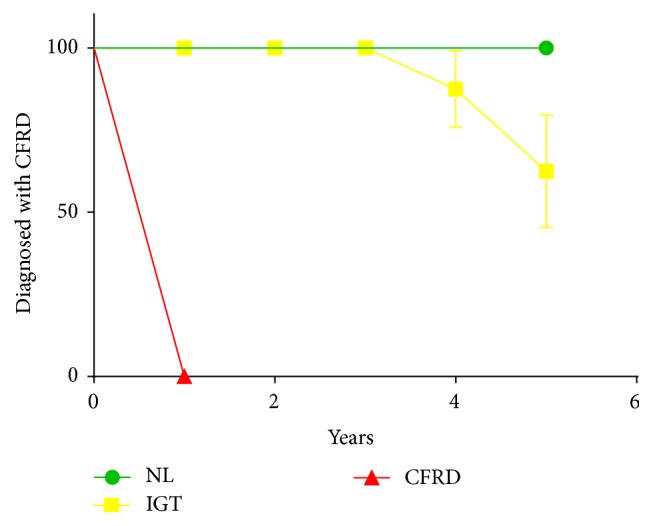
Abnormal CGM results predict the development of guideline criteria diagnosed glucose abnormalities over time. CGM results were categorized as normal (green circles), impaired (yellow squares), or consistent with CFRD (red triangles). Time to event was analyzed using the* Log-rank (Mantel-Cox) test. *0/1 subjects with normal CGM, 3/11 subjects with impaired glucose metabolism, and 5/5 subjects with CFRD by CGM were subsequently diagnosed with CFRD. (*χ* ^2^ = 17.27, DF = 2, *p* = 0.0002.)

One subject met criteria for CFRD by OGTT (as well as by the CGM criteria defined for this study: glucose > 200 mg/dL on two dates) at the time of the prospective study. Of those remaining subjects with complete CGM and OGTT data (*n* = 17), 7/17 (41%) subjects were diagnosed with CFRD within 4 years of their study enrollment (5 subjects were diagnosed by symptoms in combination with RPG > 200 mg/dL, and 2 subjects were diagnosed by subsequent OGTT) (Table 2). All of the subjects who were subsequently diagnosed with CFRD had abnormal CGM results at the time of the original study; CGM results were interpreted as consistent with IGT in 3 subjects and with CFRD in 4 subjects. One hundred percent of the subjects whose CGM results were consistent with CFRD went on to develop CFRD in the follow-up study period. Abnormal CGM (glucose > 200 mg/dL on two dates) correctly identified those subjects who would subsequently be diagnosed with CFRD (*χ* ^2^ = 17.27, DF = 2, *p* = 0.0002). See Figure 3. Two of the 7 subjects had normal OGTTs at the time of the original study; the remainder of the subjects had IFG/IGT by OGTT. One-hour glucose results were normal in 2 subjects, but the remaining 5 subjects had 1-hour OGTT results consistent with CFRD. Thus, the 1-hour OGTT result was also useful in determining which subjects would develop CFRD (*χ* ^2^ = 13.26, DF = 2, *p* = 0.001).

Of the 10 subjects who have not met criteria for CFRD since study enrollment (two of these subjects died, and 3 moved from the center), 5 subjects (50%) had at least one OGTT in the 4-5 years subsequent to study enrollment. Although 3 of 5 of the subjects had normal OGTTs at enrollment (CGM results indicated IGT in all three subjects), those 3 subjects have subsequently been diagnosed with IGT by OGTT. All 10 subjects who have not met criteria for CFRD since study enrollment have had at least one RPG and/or A1C measured in the 4-5 years subsequent to enrollment. None of the 10 subjects met criteria for CFRD based on an A1C ≥6.5% or RPG ≥200 mg/dL in conjunction with polyuria and polydipsia.

Using current glucose metabolism as the outcome (Table 2), we calculated the sensitivity, specificity, and positive (PPV) and negative predictive values (NPV) for CFRD of subjects' initial testing (CGM, 1-hour OGTT and 2-hour OGTT results). The sensitivity, specificity, PPV, and NPV of CGM for subsequent diagnosis of CFRD were 0.63 (CI 0.3–0.86), 1.0 (CI 0.71–1.0), 1 (CI 0.54–1.0), and 0.77 (CI 0.49–0.92), respectively. The sensitivity, specificity, PPV, and NPV of 1-hour OGTT for subsequent diagnosis of CFRD were 0.75 (CI 0.4–0.92), 1.0 (CI 0.69–1.0), 1 (CI 0.59–1.0), and 0.82 (CI 0.52–0.95), respectively. Finally, the sensitivity, specificity, PPV, and NPV of 2-hour OGTT for the subsequent diagnosis of CFRD were 0.13 (CI 0.03–0.48), 1.0 (CI 0.71–1.0), 1 (CI 0.15–1.0), and 0.59 (CI 0.35–0.78), respectively.

#### 3.3.2. Lung Function

There was no difference in baseline lung function between the patients who went on to develop CFRD and those who did not (67.1 ± 10.7 versus 66.3 ± 7.9, *p* = 0.95). There was a trend towards a correlation between maximum CGM values and decline in lung function over time in the group as a whole (*r* = 0.45; 95% CI −0.01 to 0.76, *p* = 0.06), but there was no correlation between these values in those later diagnosed with DM (*r* = 0.03, 95% CI −0.69 to 0.72, *p* = 0.9416).

#### 3.3.3. BMI

There was no difference in baseline BMI between the patients who went on to develop CFRD and those who did not (20.80 ± 1.08 versus 22.83 ± 1.13, *p* = 0.22). There was no correlation between maximum CGM and decline in BMI over time in the group as a whole (*r* = 0.33, 95% CI −0.16–0.69, *p* = 0.18), although there was a trend towards decline in BMI in those later diagnosed with CFRD (*r* = −0.67, 95% CI −0.93 to 0.07, *p* = 0.07).

#### 3.3.4. Pulmonary Exacerbation

There was no difference between rate of exacerbations in subjects who went on to develop CFRD versus those who did not (1.21 ± 0.29 versus 1.71 ± 0.26, *p* = 0.22). There was no correlation between maximum CGM and rate of exacerbations in the group as a whole (*r* = 0.34, 95% CI −0.70 to 0.15, *p* = 0.16), nor in those who later were diagnosed with CFRD (*r* = 0.59, 95% CI −0.20 to 0.91, *p* = 0.12).

## 4. Discussion

The goal of the prospective pilot study was to compare the ability of the gold standard OGTT versus CGM to evaluate glucose metabolism in CF. CGM detected* a greater level of impairment* than the guideline-recommended OGTT. Subsequently, we sought to determine if glucose abnormalities detected by CGM predicted decline in health. In our small study population, abnormal CGM results were not associated with decline in lung function or BMI or increased rate of exacerbations over the 5 years following the study. However, abnormal glucose metabolism (glucose > 200 mg/dL on two dates) identified by CGM accurately identified those who would later be diagnosed with CFRD. The sensitivity of CGM was similar to that of 1-hour OGTT, and both were better than that of 2-hour OGTT for subsequent development of CFRD.

CFRD occurs in approximately 40–50% of adults with CF [1, 4]. Numerous studies have shown that development of CFRD is associated with lung function and BMI decline [4, 8, 9, 20]; these adverse health effects can begin in the years prior to diagnosis [21]. A recent review of diabetes-related mortality in CF showed that the overall mortality for patients with CFRD was 1.8 per 100 person-years, compared with 0.5 in patients with CF without diabetes (*p* = 0.0002) [11]. Importantly, the increased morbidity and mortality can be improved with use of insulin therapy in patients with CFRD [4, 21–27].

Very few patients with CF have completely normal glucose metabolism [12]. In addition to those patients who are diagnosed with CFRD, 10–20% of patients with CF who do not meet criteria for diagnosis of CFRD have impaired glucose tolerance when it is evaluated [1]. Other investigators have shown that patients with elevated 1-hour OGTT values are at increased risk of developing CFRD [28, 29]. Furthermore, patients with one-hour OGTT glucose greater than 140 mg/dL have been shown to be at risk for lung function decline directly related to the degree of one-hour glucose elevation [30]. While treatment of patients with IGT is not well studied, 2 case series have shown reversal of decline in lung function and BMI decline with use of insulin therapy in CF patients with IGT [31, 32]. Thus, impaired glucose metabolism is a prevalent issue leading to increased morbidity and mortality that can be reversed with treatment when it is diagnosed.

Although rates of screening for CFRD using OGTT have increased since the guidelines [12] were published, rates of screening are still quite low. In 2014, only approximately 54% of children between 10 and 17 years of age and 28% of adults ≥18 years of age underwent CFRD screening with the recommended OGTT [1]. At our center in 2014, approximately 36% of patients ≥10 years of age underwent OGTT screening.

Because of the difficulty in obtaining annual screening OGTT, it would be ideal to use a sensitive test that does not require fasting (in a patient population that suffers from malnutrition) and an additional 2-3 hours in clinic. Although AIC is quick and easy to assess, it is not recommended for CFRD screening because it demonstrates a low degree of correlation with OGTT and is insensitive for the diagnosis of CFRD [33, 34]. In contrast to the performance of A1C against OGTT, we showed a strong correlation with CGM and 1-hour OGTT and a moderate degree of correlation with 2-hour OGTT results. CGM detected a greater degree of glucose excursions >140 mg/dL than OGTT. Because there are no dietary restrictions during CGM, CF patients' recommended diet will often exceed the 75-gram load of OGTT. However, a glucose >140 mg/dL occurs very rarely in nondiabetic individuals tested by CGM [35]. Furthermore, the test can be performed with relative ease, with a small increase in overall cost of diabetes screening.

O'Riordan and colleagues sought to validate the use of CGM in a prospective cohort of children and adolescents with CF [14]. They demonstrated that CGM performed on two occasions over a 12-month period was reliable when compared with OGTT (Bland-Altman agreement 0.81 mmol/L; 95% CI for bias ± 2.90 mmol/L), as well as reproducible and repeatable between visits. Nineteen of the twenty-one patients in our study had adequate data for analysis.

Subsequent to the validation of CGM in children and adolescents with CF, other researchers have examined whether abnormal glucose metabolism by CGM predicts clinical outcomes. Hameed et al. prospectively studied 33 children with CF who were scheduled to undergo OGTT as part of routine health screening. Twenty-five of the children agreed to simultaneously undergo CGM. They found that the amount of time a patient experienced elevated blood glucose, specifically a blood glucose of >7.8 mmol/L (140.4 mg/dL) for ≥4.5% of the time, was associated with declining nutritional status and lung function in the preceding 12 months [15]. Additionally, in a single center cross-sectional study in stable adult and pediatric patients with CF undergoing routine annual OGTT, investigators performed CGM and evaluated the effects of CGM glucose >11 mmol/L (198 mg/dL) on health outcomes. Patients with CGM glucose >11 mmol/L had lower lung function and increased prevalence of* Pseudomonas aeruginosa* lung infection [16]. These two studies show that early glucose abnormalities are detectable by CGM in children and adults with CF and are associated with historical decline and contemporaneous worse health outcomes. Finally, Schiaffini et al. [17] obtained OGTT and CGM on 17 children (mean age approximately 13 years) with CF followed by repeat OGTT in 2.5 years. They found that altered glucose metabolism measured by CGM was the best predictor of future glucose metabolism abnormalities. These studies did not answer the question of whether abnormal CGM predicts future health outcomes in adults with CF. We demonstrated that abnormal CGM does identify those who will develop CFRD. We were unable to show that maximum CGM value predicted declines in lung function and/BMI or increased rate of exacerbations. Because this study was a pilot study, it was underpowered to detect such differences. 

*Limitations*. Although OGTT and CGM were collected prospectively, information regarding subsequent diagnosis of CFRD, lung function, and BMI was collected retrospectively. In the original study, CGM data was interpreted in a blinded fashion; however, knowledge of CGM results might have led to bias/more sensitivity to symptoms by clinicians. On the other hand, abnormal OGTT results (i.e., impaired fasting glucose and/or impaired glucose tolerance) should also alert clinicians/caregivers to increase monitoring of a patient's glucose metabolism.

Six of the patients began CFTR modulators during the retrospective observational study period. Although a formal study of the impact of ivacaftor on glucose metabolism is underway (NCT02039986), to date only a case report and a case series have been published [36, 37]. Based on these reports, it is possible that CFTR modulation may have improved glucose metabolism in our six patients who received them, thus influencing their current state of glucose metabolism.

## 5. Conclusions

In adult patients with CF, CGM identified a greater degree of impaired glucose metabolism than the gold standard 2-hour OGTT. Furthermore, glucose >200 mg/dL on two dates by CGM correctly identified subjects who developed CFRD over time. The sensitivity and negative predictive values were similar for both CGM and 1-hour OGTT for subsequent development of CFRD, and both exceeded those values for the traditional OGTT measurements. Patient tolerance of CGM is excellent [14–17]. Our findings suggest that either including a 1-hour OGTT measurement or using CGM* may* provide a better tool for identifying patients who are at highest risk for the development of CFRD. Larger studies are required to test this hypothesis and to more accurately evaluate the ability of CGM data to predict changes in clinical endpoints such as lung function, nutritional status, and rate of pulmonary exacerbations. Impaired glucose metabolism is clearly associated with poor health outcomes in patients with CF; therefore, such investigation may be important as we look for better ways to quickly identify and intervene in subjects at greater risk of clinical decline.

## Figures and Tables

**Table 1:** Subject demographics at enrollment.

Variable	Enrolled	Complete CFRD testing available
	*N* = 21	*N* = 18^*∗*^
Age (years)	32.4 SD 13.1	33.8 SD 13.7
Female	76% (16/21)	72% (13/18)
Homozygous for F508del	52% (11/21)	44% (8/18)
At least one copy of F508del	90% (19/21)	89% (16/18)
FEV_1_ (L)	2.27 SD 0.84	2.23 SD 0.21
FEV_1_% predicted	68.8 SD 5.5	66.7 SD 6.3
BMI	21.6 SD 0.72	21.9 SD 0.81

^*∗*^There were no significant differences for any of the variables between the two groups.

**Table 2:** Original and current glucose metabolism.

	Original glucose metabolism interpretation			Current glucose metabolism
	OGTT	1H OGTT	CGM	Status^*∗*^
1	Normal	Normal	IGT	Normal
2	Normal	IGT	IGT, IFG	Normal
3	Normal	IGT	IGT	Normal
4	Normal	IGT	IFG, IGT	Normal
5	Normal	Normal	IFT, IGT	Normal
6	Normal	Normal	IGT	IGT
7	Normal		IGT	IGT
8	Normal	IGT	IFG, IGT	IGT
9	Normal	Normal	IFG, IGT	CFRD
10	Normal	Normal	IGT	CFRD
11	IFG	Normal	Normal	Normal
12	IGT	IGT	IGT	IGT
13	IGT	CFRD	IGT	CFRD
14	IFG	CFRD	CFRD	CFRD
15	IFG	CFRD	CFRD	CFRD
16	IGT	CFRD	CFRD	CFRD
17	IGT	CFRD	CFRD	CFRD
18	CFRD	CFRD	CFRD	CFRD

^*∗*^Current glucose metabolism status was determined by most recent OGTT when available (8 subjects) or by alternative guideline-recommended criteria for CFRD: most recent A1C of >6.5% or RPG >200 mg/dL with symptoms. For CGM, the following criteria were used for interpretation: fasting glucose >126 mg/dL or random glucose >200 mg/dL indicated CFRD; random glucose >140 mg/dL indicated IGT; fasting glucose >100 mg/dL indicated IFG.

## Abbreviations

ATS: American Thoracic Society
BMI: Body mass index
CGM: Continuous glucose monitoring
CF: Cystic fibrosis
CFF: Cystic Fibrosis Foundation
CFRD: Cystic fibrosis related diabetes
CF TDN: Cystic Fibrosis Therapeutics Development Network
CFTR: Cystic Fibrosis Transmembrane Conductance Regulator
FEV1: Forced expiratory volume in 1 second
A1C: Hemoglobin A1C
IFG: Impaired fasting glucose
IGT: Impaired glucose tolerance
NPV: Negative predictive value
OGTT: Oral glucose tolerance test
PPV: Positive predictive value
RPG: Random plasma glucose.
